# Novel T Follicular Helper-like T-Cell Lymphoma Therapies: From Preclinical Evaluation to Clinical Reality

**DOI:** 10.3390/cancers14102392

**Published:** 2022-05-12

**Authors:** Adrien Krug, Gamze Tari, Aymen Saidane, Philippe Gaulard, Jean-Ehrland Ricci, François Lemonnier, Els Verhoeyen

**Affiliations:** 1 Université Côte d’Azur, INSERM, C3M, 06204 Nice, France; adrien.krug@etu.univ-cotedazur.fr (A.K.); aymen.saidane@etu.univ-cotedazur.fr (A.S.); jean-ehrland.ricci@univ-cotedazur.fr (J.-E.R.); 2 Univ Paris Est Créteil, INSERM, IMRB, 94010 Créteil, France; gamze.tari@inserm.fr; 3 Département de Pathologie, AP-HP, Groupe Hospitalo-Universitaire Chenevier Mondor, 94010 Créteil, France; philippe.gaulard@aphp.fr; 4 Service Unité Hémopathies Lymphoides, AP-HP, Groupe Hospitalo-Universitaire Chenevier Mondor, 94010 Créteil, France; francois.lemonnier@aphp.fr; 5 CIRI, Université de Lyon, INSERM U1111, ENS de Lyon, Université Lyon1, CNRS, UMR 5308, 69007 Lyon, France

**Keywords:** AITL, PTCL, immunotherapy, RHOA, TET2, IDH2, CAR T, NF-κB, PD-1, clinical trial

## Abstract

**Simple Summary:**

This work reviews the multiple efforts that have been and are being invested by researchers as well as clinicians to improve the treatment of a specific T-cell lymphoma called follicular helper peripheral T-cell lymphoma. Still, though treatments for B-cell lymphomas have improved, this particular T-cell lymphoma has little to no new therapeutic options that show marked improvements in the survival of the patients compared to treatment with chemotherapy. We report here the evaluation of targeted new therapies for this T-cell lymphoma in new preclinical models for this cancer or in clinical trials with the objective to offer better (combination) treatment options.

**Abstract:**

The classification of peripheral T-cell lymphomas (PTCL) is constantly changing and contains multiple subtypes. Here, we focus on Tfh-like PTCL, to which angioimmunoblastic T-cell lymphoma (AITL) belongs, according to the last WHO classification. The first-line treatment of these malignancies still relies on chemotherapy but gives very unsatisfying results for these patients. Enormous progress in the last decade in terms of understanding the implicated genetic mutations leading to signaling and epigenetic pathway deregulation in Tfh PTCL allowed the research community to propose new therapeutic approaches. These findings point towards new biomarkers and new therapies, including hypomethylating agents, such as azacytidine, and inhibitors of the TCR-hyperactivating molecules in Tfh PTCL. Additionally, metabolic interference, inhibitors of the NF-κB and PI3K-mTOR pathways and possibly novel immunotherapies, such as antibodies and chimeric antigen receptors (CAR) directed against Tfh malignant T-cell surface markers, are discussed in this review among other new treatment options.

## 1. Introduction

Peripheral T-cell lymphomas (PTCL) are a diverse group of overall rare and aggressive lymphomas that develop from the oncogenic transformation of mature (i.e., post-thymic) T cells. PTCLs account for less than 10% of non-Hodgkin lymphomas worldwide. The occurrence of the different subtypes is affected by several factors, such as age, gender, ethnic origin, genetics and immunological disorders. The last WHO Classification of Tumors of Haematopoietic and Lymphoid Tissues recognized 30 distinct PTCL subtypes, which are grouped according to their main clinical presentations [1].

Among these various PTCL subtypes, T follicular helper (Tfh) PTCLs have been recently recognized as the most frequent PTCL entity in European countries, encompassing more than 30% of non-cutaneous PTCL [2,3].

Angioimmunoblastic T-cell lymphoma (AITL) is the most frequent and was the first Tfh PTCL identified. Of importance, PTCL and, thus, AITL remain rare cancers compared to B-cell lymphomas. This is an aggressive disease with poor clinical outcomes, the 5-year overall survival being around 30% and preferentially affecting patients over the age of 60. It is a systemic disease frequently associated with generalized lymphadenopathy, hepatosplenomegaly, cutaneous rash and serous involvement. Hypergammaglobulinemia and autoimmune disorders, such as autoimmune cytopenia, are frequent manifestations and could reflect the Tfh derivation of this lymphoma [4]. At the pathological level, AITL is characterized by a polymorphic infiltrate that usually destroys the lymph node architecture, composed of neoplastic T cells with a Tfh phenotype and a prominent tumor microenvironment (TME). This microenvironment is a mix of reactive hematopoietic cells, including T cells, plasma cells, eosinophils, B cells and immunoblasts, which are large B cells that are frequently EBV-positive; and stromal cells, including post-capillary venule hyperplasia and follicular dendritic cell expansion [5]. Neoplastic T cells have irregular nuclei and clear cytoplasm. They are CD4+ T cells, and they express the Tfh-associated molecules CD10, C-X-C motif chemokine ligand 13 (CXCL13), B-cell lymphoma 6 (BCL6), programmed death-1 (PD-1), C-X-C motif chemokine receptor 5 (CXCR5) and inducible co-stimulatory (ICOS) molecules [6]. Gene expression studies showed an enriched Tfh signature in AITL that resembled their normal Tfh counterparts [7].

In addition to AITL, follicular PTCL, a rare PTCL subtype where neoplastic cells have a follicular pattern and a part of PTCL not otherwise specified (PTCL-NOS), representing around 25% of PTCL NOS, expresses Tfh markers. AITL, follicular PTCL and nodal PTCL with a Tfh phenotype present similar clinical presentations, gene expression profiles, DNA copy numbers, anomalies and mutational profiles, suggesting they are closely related [8]. These three subsets were gathered under the same umbrella entity Tfh PTCL in the latest WHO classification [1].

## 2. Tfh PTCL Oncogenesis and Mutational Landscape

The development of sensitive sequencing techniques allowed a better understanding of the molecular mechanisms underlying the Tfh PTCL oncogenic transformation [9]. Tfh PTCL have a homogeneous mutational landscape, with frequent alterations in genes regulating DNA methylation/hydroxymethylation and T-cell signaling, T-cell receptor (TCR) and co-stimulation pathways [10]. Below, we will list some of these epigenetic modifiers that carry mutations in Tfh PTCL.

The tumor suppressor gene Tet Methylcytosine Dioxygenase 2 (TET2) encodes for an α-ketoglutarate and iron-dependent dioxygenase that has a critical role in 5-hydroxymethyl cytosine formation [11] via the oxidation of 5 methylcytosine (5mC). DNA methyltransferase 3 alpha (DNMT3A) is a DNA methyltransferase involved in the conversion of 5-cytosine to 5-methylcytosine [12] via methylation of cytosine. Both enzymes are frequently mutated in AITL. Isocitrate dehydrogenase NADP(+) 2 (IDH2) is also often mutated, specifically at the R172 residue, which results in a mutant with a new enzymatic activity that produces an oncometabolite, the D-2 hydroxyglutarate that inhibits many dioxygenases, including TET2 [13]. These genes are involved in epigenetic regulation, and their mutations result in 5-hydroxymethylcytosine loss through a common mechanism in PTCL [14]. TET2 and DNMT3A mutations are also frequently described in myeloid neoplasm and in clonal hematopoiesis of undetermined potential (CHIP) [15], suggesting they are not able per se to fully drive oncogenic transformation and require additional events.

In Tfh PTCL, TET2 and DNMT3A mutations are detected in neoplastic T cells and also in various cell subsets, such as CD34-derived colonies, CD34+ cells [16] and B cells isolated from AITL biopsies. This suggests that TET2 and DNMT3A mutations occur in a hematopoietic progenitor as the first hit in oncogenesis and that they could affect the function of their mutated reactive cells in the TME [17].

The Ras homolog gene family member A (RHOA) gene presents a missense mutation in 50–70% of AITL patients. RHOA is a GTPase enzyme, and the most common mutation, RHOA^G17V^, adversely affects the polarization and migration of T cells and T-cell receptor engagement [18,19,20]. Though the precise role of T-cell transformation remains unclear, RHOA mutations tend to frequently occur together with TET2 mutations. Additionally, RHOA and IDH2 mutations are restricted to tumor cells [21], indicating that they are likely the second hit in a multistep oncogenic process. These mutations are equally present in Tfh PTCL, with the exception of the IDH2^R172^ mutation, which is closely related to the AITL signature and is associated with a peculiar pathological presentation [13,21], while SYK fusions are more frequently detected in follicular PTCL [22]. Furthermore, multiple mutations altering the TCR or co-stimulation pathways have been described [10,23] and are listed below in Section 7.

## 3. Recently Developed Mouse Models Recapitulating AITL Malignancy 

Some early genetic models, such as the IDH2 R172K mutated mouse model or the Swiss Jim Lambert (SJL) mouse model, were generated in order to mimic Tfh PTCL, but only a few features of the disease were recapitulated in these models, and they are reviewed elsewhere [17]. Here, we focus on the most recent AITL-like preclinical models.

More recently, the coexistence of TET2 and RHOA(G17V) mutations in AITL led the researchers to focus on combining these mutations in the same mouse model. Zang et al. developed a Tet2^−/−^RHOA(G17V) mouse model by overexpressing the RHOA(G17V) mutant via retroviral transduction in T cells and transferring them into Tet2 knockout mice. The survival of Tet2^−/−^RHOA(G17V) mice was radically reduced compared to WT, Tet2^−/−^ or RHOA(G17V) models. Tet2-mutated and RHOA(G17V)-expressing CD4+ T cells had a higher proliferating capacity and lower apoptosis and cell death. Some key AITL features, such as lymph nodule growth, reactive cell infiltration in the organs, increased B cells in the germinal center and increased inflammatory cytokine levels (e.g., IL6), were detected in this model [24].

The awareness that Tet2 and RHOA(G17V) mutation coexistence in AITL was important led to further model developments. Cortes et al. generated a Tet2-inactivating and RHOA(G17V)-overexpressing mouse model by the transfer of Tet2^−/−^ hematopoietic progenitors into RHOA(G17V)-mutated mice. The induction of the double mutation in this mouse model resulted in lymphoma, including splenomegaly, disrupted nodal structure and follicular dendritic cell expansion in the microenvironment. Tfh hyperproliferation and Tfh signature gene enrichment were confirmed in the lymphoma of these mice. These authors distinguished an increase in the PI3K-mTOR signaling pathway related to the induction of ICOS expression in Tfh cells proving the dependency of AITL on this pathway [18].

Another Tet^−/−^ RHOA(G17V)-expressing mouse model was developed by Ng et al. A significant increase in Tfh-marker expressing cells and an enrichment in the Tfh gene signature were detected in transgenic mice compared to wild-type. These mice showed splenomegaly, lymphadenopathy, high vascularization and enlarged lymph nodes. The introduction of the RHOA(G17V) mutation exclusively in CD4+ T cells in the transgenic mice caused humoral autoimmunity, which was detected by increased IgG deposition in the kidneys, similar to human AITL. An increase in the mTOR pathway similar to Cortes’s model was detected in this model as well. Of interest, Ng et al. treated the mice with the mTOR inhibitor molecule Everolimus, and this resulted in an elongated life span of AITL-like mice [25]. For a side by side comparison between the different genetic AITL mouse models, we refer to a another recent review [17].

This highlights the required cooperation between events affecting the epigenetic landscape and signaling pathways that drive the AITL tumor transformation.

## 4. Current Treatment Strategies for AITL and Tfh PTCL Patients

The conventional first-line therapy of AITL is rooted in a chemotherapy called CHOP (cyclophosphamide, hydroxydaunorubicin, oncovin and prednisone) with additional etoposide chemotherapy in some cases. The complete response rate with CHOP-like therapies remains low, around 40% [2]. Various molecules have been tested in addition to the CHOP backbone, aiming to improve Tfh PTCL prognosis. Two phase 2 clinical trials dedicated to AITL patients have been sponsored by the French LYSA group. The first one combined the anti-CD20 monoclonal antibody rituximab with CHOP, aiming to target B cells in the microenvironment and the T/B crosstalk that could favor neoplastic cell maintenance but failed to improve the response rate compared to historical series [26]. The second one combined lenalidomide with CHOP and also did not meet the primary objective [27]. Another phase II study combining bevacizumab, an anti-VEGF (vascular endothelial growth factor) antibody with CHOP was not demonstrating improved efficacy. 

Alemtuzumab, an anti-CD52 antibody, in combination with CHOP was compared to CHOP in the ACT-2 trial. Although the response rate was higher in the alemtuzumab CHOP arm, no difference in outcome was observed, probably due to increased toxicity. [28]. The randomized phase III Ro CHOP study compared romidepsin CHOP to CHOP in untreated PTCL patients and failed to demonstrate increased progression-free survival in patients receiving the romidepsin CHOP combination [29]. However, of note, a post-hoc analysis of clinical trials showed that the addition of romidepsin might be beneficial for the AITL subgroup in this study. However, this result is, for the moment, a tendency and is not significant [30]. Conversely, the ECHELON 2 trial demonstrated an improved survival in PTCL patients with CD30-positive PTCL receiving an upfront combination of brentuximab vedotin and CHP compared to CHOP. However, a subgroup analysis suggested the absence of benefit of brentuximab vedotin addition in AITL patients. 

In total, although unsatisfactory, CHOP alone or combined with etoposide treatment is still the first-line chemotherapy in Tfh PTCL, which makes us face the hard reality that new combinations or alternative treatments are urgently needed. An extensive listing of the drugs being tested in ongoing clinical trials including Tfh PTCL and AITL patients is reviewed elsewhere [31].

Novel treatments that are being or could be considered, such as immunotherapies and inhibitors of molecules shaping the epigenetic landscape, are summarized below (Section 7).

## 5. Targeting Epigenetic Regulators Is an Emerging Concept in AITL Treatment 

**Figure 1:**
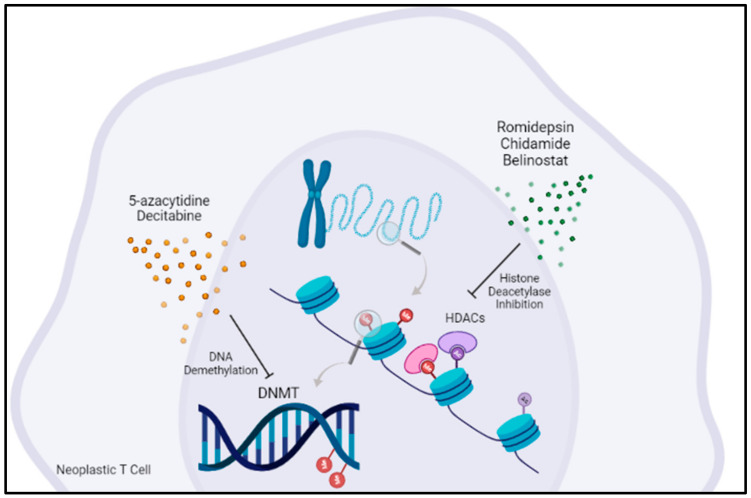
Epigenetic regulators can be targeted via several inhibitors in AITL treatment. DNMT: DNA methyltransferase; HDACs: Histone deacetylases.

Romidepsin is a histone deacetylase inhibitor that demonstrated efficacy in relapsed/refractory PTCL [32]. Chidamide is another histone deacetylase inhibitor that was combined with CHOEP therapy in a phase Ib/II clinical trial for untreated PTCL patients (Figure 1). However, no benefit of Chidamide was observed in AITL patients [33]. Similar results were obtained from another histone deacetylase inhibitor, Belinostat, in a phase II clinical trial for relapsed and refractory PTCL patients [34]. Recent data suggest that Tfh PTCL could have a better outcome when treated with HDAC inhibitors than other PTCLs [35]. However, these inhibitors also induce the deacetylation of non-histone proteins, and as such, combinations with other epigenetic targeting agents are logical but may not necessarily result in an extra benefit for the patient.

5-azacytidine is a DNMT inhibitor that acts as a hypomethylating agent. It has been approved for myelodysplastic syndrome (MDS), chronic myelomonocytic leukemia (CMML) and acute myeloid leukemia (AML), neoplasms sharing with Tfh PTCL mutations in TET2, DNMT3A and IDH2. In a retrospective study including 12 patients, 9 patients had responses, including 6 complete responses [36]. This promising result supported the initiation of an international phase III clinical trial (ORACLE: NCT03593018) comparing the efficacy and safety of oral azacytidine with investigator choice between romidepsin, bendamustine or gemcitabine. 5-azacytidine has been combined with CHOP therapy in phase II clinical trials for PTCL patients with promising results; the complete response rate in Tfh PTCL was 88% [37]. 5-azacytidine was also combined with romidepsin in first-line or relapsing PTCL patients with promising response rates [38]. Duvelisib is a PI3Kγδ inhibitor that targets the PI3K-mTOR pathway. Palomero et al. showed a decrease in tumor burden, splenomegaly rate and tumor cell proliferation and increased apoptosis in a Tet2^−/−^RHOAG17V mouse model treated with Duvelisib [20]. The results from clinical studies are encouraging, although limited by toxicity [39,40]. Furthermore, Duvelisib has been combined with romidepsin with increased efficacy and better tolerance

## 6. The NF-κB Pathway Revealed as Therapeutic Target in a New AITL Preclinical Mouse Model

Cellular metabolism plays an important role in the development of tumor cells as well as immune cells. Thus, to more precisely evaluate the role of metabolism on T-cell maturation in vivo, Mondragon et al. decided to overexpress glyceraldehyde 3-phosphate dehydrogenase (GAPDH) under the T-cell-specific promoter of LcK kinase (pLcK-GAPDH) [41]. This enzyme is known for its glycolytic and non-glycolytic activities [42,43]. Surprisingly, from the age of 18 months onwards, these mice started to develop clinical features such as skin rash and enlargement of the spleen, liver and lymph nodes. Moreover, inflammatory cytokines were increased in these tumors, and the mice showed abdominal ascite accumulation [41]. At a cellular level, organ architecture was highly altered with a high infiltration of T (essentially CD4^+^), B and dendritic cells. This phenotype directed the authors toward a potential PTCL development. First, the enlarged tissues had a strong infiltration of CD4^+^ cells that present a Tfh phenotype: high expression of Bcl6, PD-1, CXCR5, its ligand, CXCL13, and ICOS. It is known that healthy Tfh cells interact with B cells in the germinal center (GC), permitting their survival and their differentiation into plasmocytes (antibody-secreting B cells). Accordingly, the plck-GAPDH mice showed in their tumor tissues B-cell infiltrates that exclusively demonstrated a GC phenotype (high expression of FAS and GL-7). As expected, this phenotype was linked with increased levels of antibodies in the mouse sera. The Tfh T cells in the plck-GADPH mice presented a clonal or oligo-clonal T-cell receptor repertoire, confirming that this was a T-cell lymphoma. All these clinical and phenotypic features indicated a very strong resemblance with human AITL. Although the key mutation found in the GTPase RhoA in AITL patients (RHOA^G17V^) was not found in the plck-GAPDH mice, a similar mutation was found in this protein (RHOA^T37M^), with equivalent Rhoa^G17V^ function, namely, the inactivation of its binding to GTP, which favored the growth of tumor cells [18,25]. Other frequent mutations present in patients in epigenetic modifiers (e.g., TET2, DMT3 and IDH2) were not yet detected in the plck-GAPDH mouse model. In summary, the overexpression of GAPDH in T cells induces, at a certain time point, a clonality of the TCR in a subpopulation of Tfh cells and mutations permitting the outgrowth of the tumoral cells.

**Figure 2:**
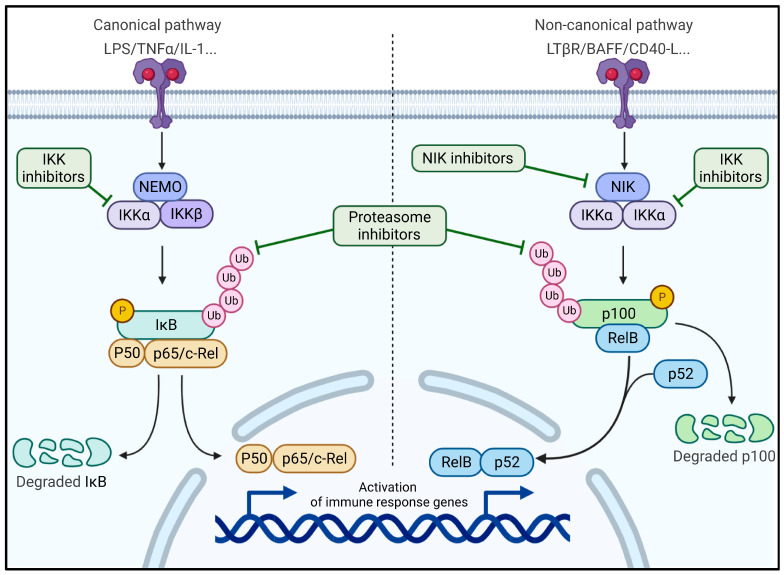
Canonical and non-canonical NF-κB pathways. Plck-GAPDH mice initially upregulate the canonical NF-κB pathways (**left**) in the CD4+ T cells before disease symptoms are evident. This activation persists for 18–24 months and induces an inflammatory environment. When the mice develop lymphoma, the tumoral CD4 Tfh cells upregulate the non-canonical NF-κB pathway (**right**). This was confirmed by the upregulation of NIK in Tfh lymphoma cells, which are accompanied by GC B cells in which NIK is also upregulated. Several inhibitors of the NF-κB pathways are indicated and might represent a new therapeutic option for AITL. Figure generated by Biorender.com.

Since GAPDH can bind TRAF2, a protein implicated in the activation of the canonical nuclear factor kappa-light-chain-enhancer of activated B cells (NF-κB pathway), this interaction might be at the origin of the chronic inflammatory environment detected in the plck-GAPDH mice (Figure 2). However, once these plck-GAPDH mice developed AITL, the non-canonical NF-κB pathway was upregulated, and this was confirmed in AITL patient tumors by gene expression analysis [41,44]. According to the authors, GAPDH could chronically activate the canonical pathway, finally inducing an activation of the non-canonical NF-κB pathway (Figure 2). To prove this hypothesis, the team developed a genetic activation of the canonical NF-κB pathway by knocking out a key effector of the pathway (IkB) in plck-IkB^−/−^ mice, which finally led to an activation of the non-canonical pathway. They also showed that the inactivation of the canonical pathway abolished CD4+ T-cell differentiation towards a Tfh phenotype. Therefore, this pathway could be a potential therapeutic target for this pathology.

NF-κB-inducing kinase (NIK) plays an important role in the differentiation and development of B cells and helper T cells [45,46,47] and activates the non-canonical NF-κB pathway through IKK phosphorylation (Figure 2) [48]. Therefore, NIK was identified as a therapeutic target in several studies, essentially as a treatment for metabolic and inflammatory diseases, but it was not considered as a treatment option for cancer until recently [49]. In the plck-GAPDH mice, which demonstrated strong activation of the non-canonical NF-κB pathway, treatment with a novel small-molecule inhibitor of NIK resulted in much lower tumor development and in a higher survival rate (patent PCT/EP2017/067306). Additionally, it is important to underline that NIK is also highly expressed in GC B cells that have a crucial role in the survival of Tfh cells as well as tumoral Tfh cells in the case of AITL. Another strategy used by the team was an immunotherapy using an anti-PD-1 antibody to target the tumoral PD-1^high^ Tfh cells. Even if this showed some efficiency, the treatment was fully optimized when combined with the NIK inhibitor. For now, neither such combination nor an NIK inhibitor alone is used in a clinical trial to combat cancer. There is, however, a clear rationale to use specific NF-κB inhibitors for lymphomas such as AITL, other PTCLs and also in other solid tumors that demonstrate an upregulation of the NF-κB pathway. Of note, a French biotech company (Yukin Therapeutics, Nice, France) was founded to develop clinically relevant NIK inhibitors for cancer treatment. 

NIK is also tightly linked to other proteins of the pathway, such as the IKK encoded by NEMO that was found to suppress NIK levels [50]. TBK1 (TANK-bonding kinase 1) can also trigger NIK degradation via its phosphorylation on Ser-862 [51,52]. Thus, it could be interesting to select these proteins as potential targets. For example, Bortezomib, a protease inhibitor with NF-κB-inhibitory activity showed promising results in the treatment of adult T-cell lymphoma [53]. Clinical trials using this drug or a new drug with a similar activity are ongoing (NCT04061772 and NCT03547700). 

## 7. Dysregulation of the TCR Signaling Pathway in AITL Reveals New Treatment Options

**Figure 3:**
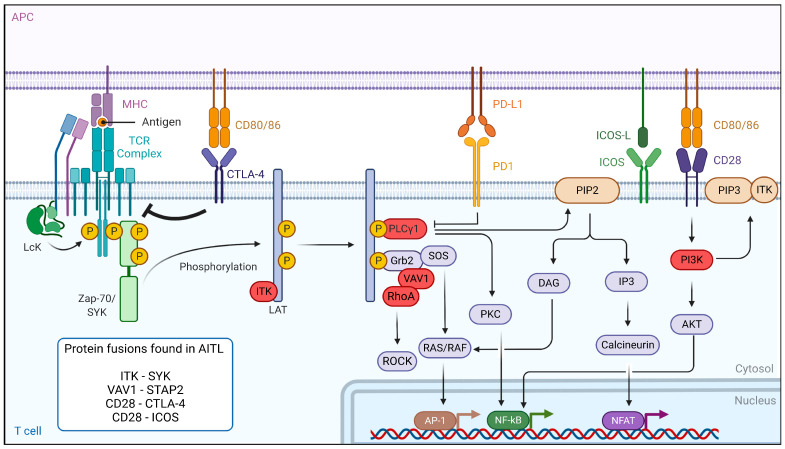
TCR-signaling and co-stimulatory pathways affected in AITL. Peptides/antigens bind to the MHC-class molecules and engage the TCR. The TCR signaling strength depends on the co-stimulatory molecules, e.g., CD28 or CTLA-4. This is followed by a series of events leading to the phosphorylation of the different components of the TCR complex. Genes mutated in the TCR-signaling pathway in AITL are indicated in red and mostly lead to antigen-independent hyper-activation of TCR signaling. Aberrant fusion proteins of the TCR signaling pathway are often encountered in AITL and are indicated in the box. Figure generated by Biorender.com.

In PTCL and AITL patients, mutations in components of the TCR signaling pathway, causing constitutive activation of the TCR, probably play a major role in T-cell pathogenesis [20,54,55]. The binding of the TCR to antigen-presenting cells leads to a cascade of events. First, the activation of the Src kinase LCK leads to the binding of zeta-chain-associated protein kinase 70 (ZAP-70) and IL-2-inducible T-cell kinase (ITK), which are then phosphorylated and activated (Figure 3). ZAP-70 then phosphorylates its targets, the adaptors linker for activation of T cells (LAT), SH2-domain-containing leukocyte protein (SLP-76) and phospholipase Cγ1 (PLCγ1), which serve as a platform for the recruitment of ITK, vav guanine nucleotide exchange factor 1 (Vav1) and the non-catalytic region of tyrosine kinase adaptor protein 1 (Nck1) in order to build a T-cell signaling complex. After its phosphorylation, PLCγ1 hydrolyzes phosphatidylinositol-4,5-biphosphate (PIP2) to produce dacylglycerol and inositol 3 phosphate (IP3), leading to the activation of nuclear factor of activated T cells (NFAT). In parallel, co-stimulation through CD28 leads to PI3K activation and PIP3 accumulation in the cells. PIP3 binds to ITK and forms a signaling complex at the cell membrane. The activation of the TCR also modulates other downstream signaling pathways, including the PI3K, NF-κB, MAPK and GTPase-dependent pathways [56].

The RHOA^G17V^ mutant is found frequently (70%) in AITL malignant cells. This mutation drives Tfh differentiation, but it can also bind Vav1, resulting in Vav1 hyperphosphorylation [57] and NFAT signaling activation, inducing T-cell proliferation and transformation [10,18,23]. This indicates that the RHOA^G17V^ mutation could be a major player in T-cell signaling activation. For this reason, multikinase inhibitors (desatinib) or PI3K inhibitors (duvelisib) are proposed as therapeutic options [19,20]. Additionally, many other components of the TCR signaling pathways, such as phospholipase C**γ**1 (14%) [10], CD28 (9–11%) [23,58], Src family tyrosine kinase (FYN) (3–4%) [10] and Vav1 itself (50%) are mutated in AITL. Moreover, a VAV1-STAP2 fusion protein is detected in some cases. To therapeutically interfere with the antigen-independent strong TCR signaling caused by these mutations, one option is to use calcineurin inhibitors, such as cyclosporine 1 [59]. Authors reviewed several clinical trials and extracted a total of 26 patients with AITL receiving cyclosporine. Interestingly, the overall response rate in AITL patients was impressive (86%), suggesting the utility of using calcineurin inhibitors in already-treated AITL cases [59]. However, the authors do insist that prudence is warranted in the interpretation and comparison of different clinical studies, especially because of the small number of AITL cases. Finally, in the case of mutated Vav1, Rac 1 inhibitors could also be an option [60].

Importantly, ITK is highly expressed in AITL patients. One study reports that ITK was highly phosphorylated in 70% of the AITL patients, and this correlated with a poor response to first-line treatment [61]. Additionally, spleen-associated tyrosine kinase (SYK), normally expressed in B cells, demonstrated an aberrantly high expression in the majority of AITL patients [22]. Therefore, SYK inhibitors might be promising therapeutic agents. In addition, translocation in some PTCL patients, including AITL, resulted in the ITK-SYK and ITK-FER fusions [22,62], which induced, independent of antigen binding, phosphorylation of TCR proximal proteins and acted as strong oncogenic drivers in T-cell lymphoma in mouse models [22,62,63]. Therefore, ITK is a target for the design of new candidates for targeted therapies, especially since it was shown that ITK inhibition leads to a decrease in the invasion and migration of malignant T cells [61,64]. This suggests that ITK inhibitor treatment might be effective in ITK-positive AITL patients since preclinical data showed anti-cancer activity in B- and T-cell leukemia [65]. Evidently, SYK inhibitors could also be proposed in the case of ITK-SYK fusion [22]. A combination of an ITK inhibitor with CHOP may be a promising therapeutic regimen. Ibrutinib was also evaluated for T-cell lymphoma since it was shown to be specific not only for BTK (Bruton tyrosine kinase) but also inhibited ITK [66,67]. Ibrutinib was tested in a clinical trial enrolling T-cell lymphoma patients. However, this only resulted in an overall response rate of 8% in these patients. Currently, CPI-818, a new selective ITK inhibitor is being tested in a phase I clinical trial including patients with refractory T-cell lymphoma, but no results are currently available (NCT03952078). 

As mentioned above, an efficient immune response relies on TCR costimulatory molcules providing a secondary signal upon TCR engagement with an antigen. CD28 is one of these co-stimulation receptors expressed at the T-cell surface and is mutated in 10% of AITL patients. Two specific CD28 mutations (D124 and T195) result in extended activation of the T-cell receptor because they have a stronger affinity for their ligand than the WT CD28 and induce signaling pathways implicated in cytokine production and T-cell proliferation [58]. Moreover, the CD28-ICOS and CD28-CTLA4 gene fusions were detected in AITL [10,68]. Unexpectedly, the CD28-CTLA4 fusion gene can convert the normal inhibitory signals induced by CTLA4 stimulation in hyperactive CD28 signaling in AITL T cells [68]. Recently, a mouse model expressing the CTLA4-CD28 fusion exclusively in a T-cell lineage was generated, and these mice developed an AITL-like lymphoma, underlining the CTLA4-CD28 transforming capacity [69]. Nguyen et al. [70], using a genetic mouse model for AITL (TET2^−/−^; RHOA^G17V^), demonstrated that dasatinib, a multikinase inhibitor, inhibited hyperactivated TCR signaling in these mice and increased their survival. Additionally, they report a clinical phase I trial including five AITL patients that confirmed a high response rate to dasatinib (UMIN000025856). These results need to be taken with care since the small number of patients limited the relevance of the study. Another phase I/II trial is ongoing using dasatinib. Importantly, the same drug also blocked the RhoA-Vav1 TCR signaling in AITL [57]. Alternatively, in the case of CTLA4-CD28, an anti-CTLA4 immunotherapy (ipilimumab) is proposed [68].

FYN is a tyrosine kinase that also plays an essential role in T-cell activation. Multiple mutations in FYN, which invalidate the inhibitory function of the FYN domain SH2 and result in a constitutive activation of the tyrosine kinase and T-cell activation [20] were identified in AITL patients. Many more mutations in PLCG1, CARD11 and CTNNB1 as well as proteins involved in the PI3K or MAPK pathways, which are implicated in the stabilization of stimulatory signals in T cells, have been described in AITL. Interestingly, CARD11 mutations seem to be at the origin of constitutive NF-*κ*B signaling and might assist in tumor outgrowth [10]. It was actually the NF-*κ*B pathway that was shown to be constitutively upregulated in one of the recent preclinical AITL mouse models (Section 6 and [41]).

## 8. Immunotherapeutic Approaches for AITL

Escaping the immune system is the main characteristic of a tumor cell. In order to limit the anarchic proliferation of cancer cells, chemotherapies are often used in tumors that have no specific treatment yet, such as AITL. These therapies are proposed to limit the proliferation of dividing cells, i.e., healthy cells as well as tumor cells. This explains the poor benefits of such treatment and the major risk of relapse or even the absence of signs of remission. Importantly, targeting a specific cancer cell type without inducing an effect on healthy cells requires an in-depth knowledge of its phenotype and its surface markers, which can serve as an asset for the development of specific therapies, in particular immunotherapies. If some healthy cells are affected, it is called on-target off-target effects because certain cancer epitopes are partially expressed on non-malignant cells. These therapies include, for example, monoclonal antibodies and chimeric antigen receptors. 

### 8.1. Monoclonal-Antibody-Based Immunotherapies for AITL Treatment 

**Figure 4:**
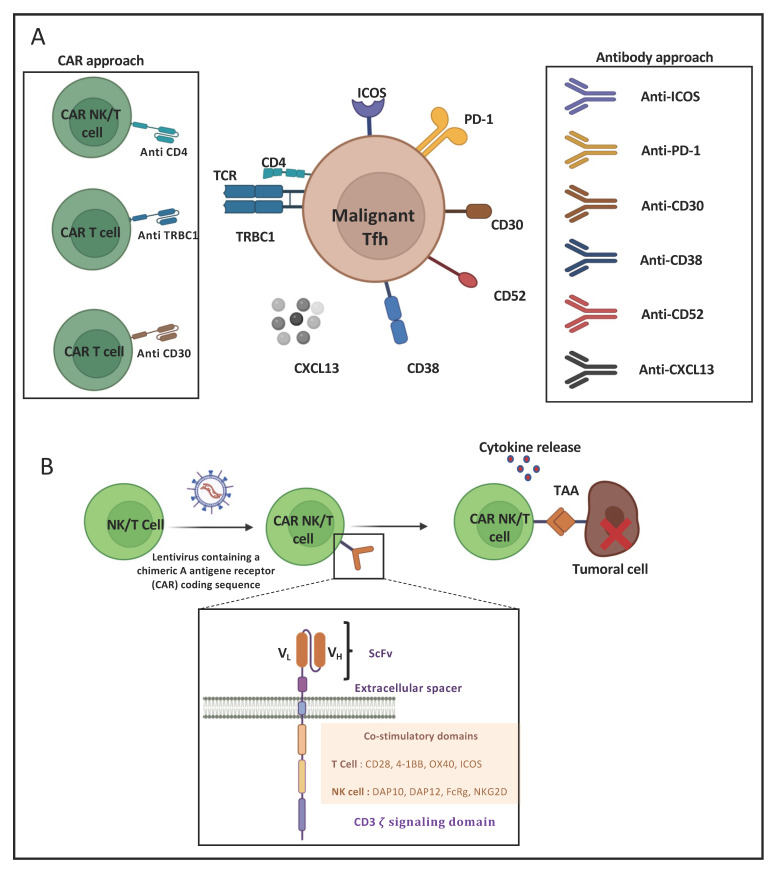
AITL immunotherapy approaches. (**A**) T-cell follicular helper (TFH) cells of AITL express several surface markers targetable by antibodies and CARs: inducible T-cell co-stimulator (ICOS), programed death 1 (PD-1), cluster of differentiation (CD) CD4, CD30, CD38, CD52 and TRBC1. Neutralizing antibodies against chemokine 13 (CXCL13) can also be used to inhibit TFH migration to germinal center. (**B**) Design of CAR-NK/T-cell generation and tumor targeting. Natural killer (NK) or T cells are transduced with a lentivirus to express a chimeric antigen receptor (CAR). NK/T cells will, via the single chain variable fragment (scFv) exposed by the CAR, recognize the tumor-associated antigen (TAA), allowing NK/T-cell activation through the CAR signaling and co-stimulation domains. VL: variable light chain. VH: variable heavy chain.

Monoclonal antibodies aim either to deplete the target by inducing antibody-dependent cell-mediated cytotoxicity, complement-dependent cytotoxicity or the direct triggering of apoptosis leading to cell death [71]. However, they can also function by blocking a cell–cell interaction or a pathway favorable to tumor cell development, proliferation or survival. The CD4+ Tfh cell, the malignant driver of AITL, highly expresses surface markers, such as CXCR5, PD-1, ICOS and CD40L, that determine its phenotype [7] and can serve as targets for immunotherapy (Figure 4A). Indeed, the CD4+ Tfh cells play an important role in B-cell maturation within the germinal center towards GC B cells. Their survival is highly dependent on ICOS surface expression, which is induced by TCR stimulation [72]. Moreover, ICOS plays a crucial role in Tfh localization [73]. Once Tfh cells leave the T zone and migrate to the germinal center, ICOS boosts the cognate T- to B-cell interaction and thereby enables an optimal antibody-based immune response [74]. AITL Tfh CD4 cells show very high similarity to healthy Tfh cells in their T-cell–B-cell interaction through ICOS-ICOSL binding. Thus, ICOS was considered a possible target for immunotherapy. Currently, a phase I clinical trial targeting ICOS by the MEDI-570 antibody is ongoing, in which recurrent AITL patients were included among other peripheral T-cell lymphomas (NCT02520791). The expected outcome of this trial is a block in Tfh growth and tumor progression, but no clear beneficial results are available, though the treatment does not seem to be toxic.

As ICOS, PD-1 is a B7 family molecule highly expressed by Tfh. While PD-1 is known to be inhibitory in immune cells, PD-1 plays an important role in Tfh development and activity. The PD-1 interaction with its ligand PDL1, which is expressed by B cells, favors the follicular recruitment of T cells expressing ICOS at the T–B border [75]. Within the germinal center, PD-1 expression controls Tfh and B-cell proliferation and survival [76]. In addition, PD-1 was found to inhibit the cytotoxic function by interacting with the PDLs expressed on anti-tumoral cells, such as natural killers and cytotoxic CD8 cells in lymphoma [77]. Taken together, PD-1 presented an important candidate target for immunotherapy in AITL. In this context, Mondragon et al. used an anti-PD-1 antibody in combination with a non-canonical NF-kB inhibitor to treat mice bearing AITL tumors. Survival increased up to 70% compared to non-treated mice [41]. These are encouraging results, but the same study showed only 40% survival upon anti-PD-1 as a single treatment. Fiore et al. [78] mentioned that pembrolizumab and nivolumab (humanized anti-PD-1 antibodies) were effective in different lymphoma subtypes. Pembrolyzumab was highly effective in relapsed or refractory NK/T-cell lymphoma patients, but blockade of the PD-1 axis is still disputable in other lymphomas subtypes [78]. It was also believed that in PTCL PD-1 itself could be a tumor suppressor [79] suggesting that anti-PD-1 could lead to exactly the opposite of what is intended, meaning increased lymphoma. Indeed, in some mouse studies, anti-PD-1 treatment caused a violent progression of adult T-cell lymphomas [80]. At present, trials including multiple types of PTCL have shown only very low activity upon anti-PD-1 as a single treatment [81,82]. A combination of checkpoint inhibitors with other agents might enhance the anti-tumor activity in T-cell lymphoma [83]. Importantly, clinical studies showed that classic HDAC inhibitors improved AITL patient ORR over 33–50% but enhanced PD-1 expression. An ongoing phase II clinical trial is testing Chilamide (HDAC inhibitor) combined with Sinlimab [84], a stable anti-PD-1 antibody (NCT04831710).

The T-cell follicular helper (Tfh) origin of AITL expresses several surface markers: inducible T-cell co-stimulator (ICOS), programed death 1 (PD-1), cluster of differentiation (CD) CD4, CD30, CD38, CD52 and TRBC1. Antibodies and CAR-NK/T cells target these extracellular markers. Indeed, a surface target was revealed in a cohort of 62 AITL patients, which showed that CD38-positive AITL cell presence was a risk factor associated with reduced overall survival [85]. An anti-CD38 antibody, Daratumuab is already approved for myeloma treatment. Moreover, a phase II clinical trial is ongoing to treat AITL patients with Daratumuab. However, it is not administered as a single agent but in combination with chemotherapies (NCT04251065). In AITL, CD30 surface expression can be detected in up to 43% of the patients [86]. CD30 is a transmembrane glycoprotein that has a pleiotropic effect on cell growth and survival [87]. Brentuximab Vedotin is an anti-CD30 antibody approved by the FDA in relapsed and refractory CD30+ PTCL, as recently the ECHELON-2 trial showed a statistical improvement in the response rate for 83% of patients. However, an AITL subgroup analysis did not indicate an improvement for these patients. Currently, a phase II clinical trial is recruiting to test Brentuximab Vedotin as a therapy for AITL patients among other lymphoma subtypes (NCT02588651). Since in AITL the vast majority of the B-cell component of the lymphoma consists of GC B cells expressing the CD20 mature B-cell marker, CD20 was considered to be an indirect target for immunotherapy in AITL. The T- and B-cell interaction in AITL is indeed important for their mutual survival. Retuximab is an antibody targeting CD20 that provided high efficacy in B-cell lymphoma [88]. Currently, a clinical trial is testing Retuximab in relapsed/refractory AITL patients combined with lenalidomide, a potent anti-angiogenesis and a neoplasm-bocking antibody (NCT04319601). Moreover, CXCR5 is highly expressed by Tfh cells in AITL and binds the chemokine CXCL13, which allows follicular homing and is a chemoattractant for B cells. The neutralizing antibody anti-chemokine 13 (CXCL13) may lead to a reduction in B-cell recruitment in the germinal center as suggested by Brodfuehrer et al. in a mouse model [89]. However, no proof of its efficacy to treat AITL malignancy has been reported. 

Finally, CD52 is considered as an immunotherapy target since it is a glycophosphatidylinositol that is widely expressed by the immune system and, thus, by both B and T cells. This cell surface protein plays an important role in T-cell homeostasis, immunosuppression and NF-*κ*B inhibition [90]. CD52 is used as target for T- and NK-cell malignancies, including AITL, in which CD52 is highly expressed [91]. Therefore, several clinical trials have been initiated using Alemtuzumab (CD52 antibody) [28,92]. Currently a clinical trial is using Alemtuzumab in addition to dose dense CHOP (A-CHOP-14) to treat PTCL, including AITL patients (NCT00725231). However, in several trials this drug treatment revealed high toxicity and does not show a clear beneficial effect. 

### 8.2. CAR-T-Cell-Based Immunotherapies for AITL Treatment 

CAR-cell therapy consists of genetically modifying a natural killer (NK) or a T cell so they can express a chimeric antigen receptor (CAR). These synthetic receptors allow the CAR-NK or -T cells to recognize a tumor-associated antigen and eliminate the cancer cells that escaped the immune system (Figure 4A,B). The CAR structure is similar to a T-cell receptor (TCR). Nowadays, five CAR generations exist, depending on their intracellular functional domains [93]. The most commonly used CAR-T cells are already accepted by the FDA for patient treatment and contain the CD28 and/or the 4-1BB co-stimulatory domains and the CD3ζ signaling chain. The choice of a co-stimulatory signal influences the T-cell metabolic status and persistence in vivo [93]. The extracellular part is composed of a single-chain variable fragment (scFv) made of a light and heavy variable region of an antibody (VL and VH) that senses and binds to a target antigen. CAR-T-cell treatment gave spectacular results in B-cell malignancies treated with anti-CD19 CAR-T cells [94]. Several clinical trials are ongoing to extend this strategy to different lymphoma subtypes. Many ongoing CAR-NK/T-cell therapies are being evaluated in clinical trials recruiting PTCL patients, including AITL, because of common surface targets. The recent generation of AITL mouse models very closely recapitulating human disease (see Section 3 and Section 5) could allow advances in the testing of CAR-T/NK-cell approaches [17]. However, it needs to be mentioned that T-cell lymphomas do not express surface markers that are excluded from healthy T cells. Therefore, the choice of the CAR-specific target is not an easy one because, as a side-effect, the CAR-T cells could attack themselves through T-cell fratricide, a mechanism originally revealed to maintain T-cell homeostasis. CAR-T cells expressing the fusion with CD3ζ acquire a specificity for ligands expressed on hematological and solid cancers. However, these ligands or receptors can also be transiently or permanently expressed by activated T cells, implying that CAR-T cells may undergo self-killing (fratricide) during CAR-T-cell production [95]. This might hinder therapeutic efficiency since T-cell fratricide might prevent the production of the required quantities of T cells for clinical applications. This is particularly true when the CAR target itself is specific for the T-cell lineage (CD4, CD7 or CD5) in order to eliminate T-cell leukemic cells. Therefore, research is conducted to avoid this unwanted effect [95,96].

AITL Tfh cells are derived from CD4 T cells. Thus, using anti-CD4 CAR-NK/T cells in AITL patients might represent a promising strategy, even though healthy CD4 T cells will also be depleted. This latter means that in the long term this approach can lead to CD4 T-cell elimination and immunosuppression. If depleting normal B cells seem to be well-tolerated in B-cell malignancies by CAR therapies, there is no sufficient insight to extrapolate this to T-cell lymphomas yet. Pinz et al. [97] constructed anti-CD4 CAR-NK that specifically eliminated robustly diverse ex vivo CD4+ human T-cell leukemia and lymphoma cell lines in vivo. These preclinical results are encouraging for anti-CD4 CAR-NK therapy use in case of all CD4+ T-cell malignancies and in particular for AITL [97]. In fact, clinical trials are ongoing using anti-CD4 CAR-T cells for relapsed/refractory T-cell lymphoma, including AITL (NCT04712864). The advantage of an anti-CD4 NK-cell application might be that NK cells are short lived in vivo compared to CAR-T cells and do not lead to extended healthy CD4 T-cell immunosuppression in the patients. However, this still needs to be consolidated.

While targeting the CD4 antigen also depletes normal CD4 T cells, M. Maciocia et al. [98] used a more specific approach to target malignant T cells. The TCR comprises a heterodimeric protein complex of two chains, TCRα and TCRβ. An ancestral duplication of the β-chain constant gene results in the expression of one of two highly homologous chains, T-cell receptor β-chain constant (TRBC) domains 1 and 2, in a mutually exclusive manner following the TCR locus rearrangement. Based on the mutually exclusive expression of TRBC1 and TRBC2, they developed anti-TRBC1 CAR-T cells that recognized and killed normal and malignant TRBC1+ but not TRBC2+ T cells in vitro and in a disseminated mouse model of leukemia. This strategy allows the selective targeting and depletion of T cells carrying the TRBC1 chain, both healthy and malignant, while sparing healthy T cells expressing TRBC2, thereby preserving T-cell-mediated immune responses [98]. A phase I/II study at the recruiting phase will evaluate AUTO4 (anti-TRBC1 CAR-T cells) in AITL patients with TRBC1 CD4+ malignant cell clonality (NCT03590574 and NCT0482817).

Finally, as previously described, CD30 is a tumor cell marker in AITL. To explore the CD30 potency as a target, clinical trials at the recruiting stage are ongoing to explore anti-CD30 CAR-T cells as a therapy to treat AITL patients and other T-cell lymphomas and leukemia (NCT04008394).

## 9. Metabolic Interference, a Future New Therapeutic Option for AITL and Tfh-like Lymphoma?

Immuno-metabolism shapes immune cell functions, and the differentiation of immune cells and interference with metabolism revealed novel anti-cancer therapies [99]. Fundamental metabolic changes are implemented by immune cells as well as cancer cells to meet their high energy requirement, for which they show increased dependence on glycolysis (Warburg effect). However, the role of specific metabolic enzymes is just starting to be unraveled. One of these enzymes, central in glycolysis, GAPDH, is only recently emerging as a key player in T-cell survival, development and function [100,101]. Surprisingly, when GAPDH was exclusively expressed in the T-cell lineage of mice, they developed a Tfh-like PTCL with a strong GC B-cell component equivalent to AITL [41]. However, as explained above (Section 5), the malignancy seems to be the consequence of GAPDH non-glycolytic functions through the activation of the NF-κB pathway, though it cannot be excluded that in early stages the role of GAPDH in glycolysis did play a role.

A major breakthrough was the discovery of the cellular derivation of AITL from T follicular helper (Tfh) cells, characterized by Bcl-6, a master regulator in Tfh differentiation [102] and GC formation [103,104]. Bcl-6 directly represses genes encoding molecules involved in the glycolytic pathway to induce their Tfh differentiation and, thus, interferes with the T-cell metabolic state [105]. In this context, it is important to mention that the Bcl-6 locus is hyper-methylated in AITL Tfh cells and that this leads to an increased/stabilized Bcl-6 expression [105,106]. Therefore, interfering with the metabolic addiction of Tfh lymphoma cells from AITL or Tfh PTCL by targeting Bcl-6 might be a therapeutic option since Bcl-6 is important for the survival of both Tfh and GC B cells. Currently, Bcl-6 is already a cancer therapeutic target in B-cell lymphomas [107,108], and Bcl-6 degraders find their way to the clinic [109]. However, the role of Bcl-6 in AITL pathogenesis is not clear, and, thus, the outcome of inhibiting Bcl-6 is not known.

Additionally, AITL Tfh cells express high levels of the hallmark immune checkpoint molecule, PD-1. High PD-1 expression was associated with the activation of T cells and also with a pronounced mitochondrial metabolism [110]. Upon PD-1 binding to its receptors PD-L1 or PD-L2, it prevents the CD28-mediated activation of PI3K and, thus, AkT, regulating in that way T-cell activation and survival [111]. PD-1 signaling also results in the reduced expression of cMyc and activity of the PI3K/Akt/mTOR pathway and inhibits glycolysis in this way [112]. PD-1 plays a major role in T-cell exhaustion. Thus, cells stimulated with PD-1 reduce their glucose uptake and use neither glycolysis nor catabolism of glutamine. Indeed, Patsoukis et al. demonstrated that PD-1 engagement in activated T cells switches their metabolism towards lipolysis and fatty acid oxidation [113]. An antibody-mediated blockage of PD-L1 reduced the tumor glycolysis rate and restored the level of glucose in the TME and consequently increased anti-cancer T-cell effector function [100]. Additionally, PD-1 was shown to inhibit peroxisome proliferator-activated receptor-gamma coactivator 1 alpha (PGC-1α), which led to loss of mitochondrial mass in TILs, a phenotype that was reverted by PGC-1α overexpression [114]. Therefore, interfering with PD-1 through immunotherapy might not only reactivate immune responses but, at the same time, fragilize the malignant Tfh cells by switching their metabolic profile. Another option might be to directly inhibit fatty acid oxidation metabolism.

It is clear that PTCL and AITL T cells acquired unique metabolic signatures that differ from healthy T cells, such a lipid metabolism in the case of PTCL-NOS [115,116] as more specific lipid pathways were also identified in T-cell lymphomas [117]. Interestingly, the reprogramming of malignant T-cell metabolism towards glycolysis and glutaminolysis was achieved by treatment with a demethylating agent [118,119]. In specific cases of AITL carrying a IDH2^R171K^ mutation, malignant T cells produce (2R)-2-Hydroxyglutarate (2HG), which inhibits α-ketoglutarate-dependent dioxygenases. In IDH2^R171K^-positive AITL patients, α-ketoglutarate derived from glutamine metabolism regulates demethylation through these enzymes in aerobic conditions, and, thus, both the TCA cycle and glutamine metabolic pathways may be affected [13]. Therefore, an inhibitor of α-ketoglutarate-dependent demethylation, e.g., S2HG facilitates the emergence of central memory CD8^+^ T cells. Alternatively, the inhibition of glutamine metabolism may decrease α-ketoglutarate and, as a result, increase a hyper-methylation state of the DNA in T cells.

In conclusion, metabolic interventions in AITL or Tfh PTCL might be considered in the future, and hopefully the newly emerged models of this rare T-cell lymphoma will allow us to identify possible metabolic targets and to evaluate novel metabolic drugs or indirect interventions that might interfere with the specific metabolic addiction of the malignant T cells.

## 10. Conclusions

AITL and Tfh PTCL are complex malignancies due to the implication of multiple cell types in the TME. Although AITL and Tfh-like PTCL are the most frequent PTCL entities, the number of patients is still not sufficient to conduct reasonable clinical trials. The Evaluation of new therapeutic options for AITL is, therefore, a challenge since this is a very rare cancer for which patient samples are not readily available and clinical trials will only include a few AITL or Tfh-like PTCL patients. Therefore, the outcome of these trials might not reveal clear-cut results in terms of therapeutic benefit for the patients. Luckily, three valid preclinical mouse models for AITL are now finally available. Today, three genetic mouse models mimic AITL cancer in terms of clinical, pathological, histological, transcriptional, genetic and immunophenotypic features: (1) two mouse models are based on the knockout of Tet2 and overexpression of RhoAG17V, (2) another model was generated by overexpression in the T-cell lineage of a key enzyme in glycolysis, GAPDH. These models allowed the discovery of signaling pathways or epigenetic players that can be tested in vivo as therapeutic targets. Already, these models were able to reveal that AITL tumors upregulated the mTOR or NF-κB pathways and enabled the testing of therapeutic options interfering with these pathways. This proves that these AITL preclinical mouse models might allow us to evaluate complex novel combination therapies and pave the way for their clinical translation. Many possible new therapeutic options, such as epigenetic modifiers, signaling pathway inhibitors or phenotypic surface markers for immunotherapy, have been identified and are being evaluated for their safety and efficacy in preclinical models and in clinical trials and might, in the near future, broaden the therapeutic options and improve the clinical outcomes of patients.

## References

[1] Swerdlow S.H., Campo E., Pileri S.A., Harris N.L., Stein H., Siebert R., Advani R., Ghielmini M., Salles G.A., Zelenetz A.D. (2016). The 2016 Revision of the World Health Organization Classification of Lymphoid Neoplasms. Blood.

[2] de Leval L., Gisselbrecht C., Gaulard P. (2010). Advances in the Understanding and Management of Angioimmunoblastic T-Cell Lymphoma. Br. J. Haematol..

[3] Laurent C., Baron M., Amara N., Haioun C., Dandoit M., Maynadié M., Parrens M., Vergier B., Copie-Bergman C., Fabiani B. (2017). Impact of Expert Pathologic Review of Lymphoma Diagnosis: Study of Patients From the French Lymphopath Network. J. Clin. Oncol..

[4] Jaffe E., Arber D., Campo E., Harris N. (2016). Quintanilla-Fend L. Hematopathology e-Book.

[5] de Leval L., Parrens M., Le Bras F., Jais J.-P., Fataccioli V., Martin A., Lamant L., Delarue R., Berger F., Arbion F. (2015). Angioimmunoblastic T-Cell Lymphoma Is the Most Common T-Cell Lymphoma in Two Distinct French Information Data Sets. Haematologica.

[6] Federico M., Rudiger T., Bellei M., Nathwani B.N., Luminari S., Coiffier B., Harris N.L., Jaffe E.S., Pileri S.A., Savage K.J. (2013). Clinicopathologic Characteristics of Angioimmunoblastic T-Cell Lymphoma: Analysis of the International Peripheral T-Cell Lymphoma Project. J. Clin. Oncol..

[7] de Leval L., Rickman D.S., Thielen C., de Reynies A., Huang Y.-L., Delsol G., Lamant L., Leroy K., Brière J., Molina T. (2007). The Gene Expression Profile of Nodal Peripheral T-Cell Lymphoma Demonstrates a Molecular Link between Angioimmunoblastic T-Cell Lymphoma (AITL) and Follicular Helper T (TFH) Cells. Blood.

[8] Dobay M.P., Lemonnier F., Missiaglia E., Bastard C., Vallois D., Jais J.-P., Scourzic L., Dupuy A., Fataccioli V., Pujals A. (2017). Integrative Clinicopathological and Molecular Analyses of Angioimmunoblastic T-Cell Lymphoma and Other Nodal Lymphomas of Follicular Helper T-Cell Origin. Haematologica.

[9] Rodríguez M., Alonso-Alonso R., Tomás-Roca L., Rodríguez-Pinilla S.M., Manso-Alonso R., Cereceda L., Borregón J., Villaescusa T., Córdoba R., Sánchez-Beato M. (2021). Peripheral T-Cell Lymphoma: Molecular Profiling Recognizes Subclasses and Identifies Prognostic Markers. Blood Adv..

[10] Vallois D., Dobay M.P.D., Morin R.D., Lemonnier F., Missiaglia E., Juilland M., Iwaszkiewicz J., Fataccioli V., Bisig B., Roberti A. (2016). Activating Mutations in Genes Related to TCR Signaling in Angioimmunoblastic and Other Follicular Helper T-Cell–Derived Lymphomas. Blood.

[11] Kohli R.M., Zhang Y. (2013). TET Enzymes, TDG and the Dynamics of DNA Demethylation. Nature.

[12] Yang L., Rau R., Goodell M.A. (2015). DNMT3A in Haematological Malignancies. Nat. Rev. Cancer.

[13] Lemonnier F., Cairns R.A., Inoue S., Li W.Y., Dupuy A., Broutin S., Martin N., Fataccioli V., Pelletier R., Wakeham A. (2016). The IDH2 R172K Mutation Associated with Angioimmunoblastic T-Cell Lymphoma Produces 2HG in T Cells and Impacts Lymphoid Development. Proc. Natl. Acad. Sci. USA.

[14] Lemonnier F., Poullot E., Dupuy A., Couronné L., Martin N., Scourzic L., Fataccioli V., Bruneau J., Cairns R.A., Mak T.W. (2018). Loss of 5-Hydroxymethylcytosine Is a Frequent Event in Peripheral T Cell Lymphomas. Haematologica.

[15] Bick A.G., Weinstock J.S., Nandakumar S.K., Fulco C.P., Bao E.L., Zekavat S.M., Szeto M.D., Liao X., Leventhal M.J., Nasser J. (2020). Inherited Causes of Clonal Haematopoiesis in 97,691 Whole Genomes. Nature.

[16] Lewis N.E., Petrova-Drus K., Huet S., Epstein-Peterson Z.D., Gao Q., Sigler A.E., Baik J., Ozkaya N., Moskowitz A.J., Kumar A. (2020). Clonal Hematopoiesis in Angioimmunoblastic T-Cell Lymphoma with Divergent Evolution to Myeloid Neoplasms. Blood Adv..

[17] Mhaidly R., Krug A., Gaulard P., Lemonnier F., Ricci J.-E., Verhoeyen E. (2020). New Preclinical Models for Angioimmunoblastic T-Cell Lymphoma: Filling the GAP. Oncogenesis.

[18] Cortes J.R., Ambesi-Impiombato A., Couronné L., Quinn S.A., Kim C.S., da Silva Almeida A.C., West Z., Belver L., Martin M.S., Scourzic L. (2018). RHOA G17V Induces T Follicular Helper Cell Specification and Promotes Lymphomagenesis. Cancer Cell.

[19] Sakata-Yanagimoto M., Enami T., Yoshida K., Shiraishi Y., Ishii R., Miyake Y., Muto H., Tsuyama N., Sato-Otsubo A., Okuno Y. (2014). Somatic *RHOA* Mutation in Angioimmunoblastic T Cell Lymphoma. Nat. Genet..

[20] Palomero T., Couronné L., Khiabanian H., Kim M.-Y., Ambesi-Impiombato A., Perez-Garcia A., Carpenter Z., Abate F., Allegretta M., Haydu J.E. (2014). Recurrent Mutations in Epigenetic Regulators, *RHOA* and *FYN* Kinase in Peripheral T Cell Lymphomas. Nat. Genet..

[21] Steinhilber J., Mederake M., Bonzheim I., Serinsöz-Linke E., Müller I., Fallier-Becker P., Lemonnier F., Gaulard P., Fend F., Quintanilla-Martinez L. (2019). The Pathological Features of Angioimmunoblastic T-Cell Lymphomas with IDH2R172 Mutations. Mod. Pathol..

[22] Streubel B., Vinatzer U., Willheim M., Raderer M., Chott A. (2006). Novel t(5;9)(Q33;Q22) Fuses ITK to SYK in Unspecified Peripheral T-Cell Lymphoma. Leukemia.

[23] Vallois D., Dupuy A., Lemonnier F., Allen G., Missiaglia E., Fataccioli V., Ortonne N., Clavert A., Delarue R., Rousselet M.-C. (2018). RNA Fusions Involving CD28 Are Rare in Peripheral T-Cell Lymphomas and Concentrate Mainly in Those Derived from Follicular Helper T Cells. Haematologica.

[24] Zang S., Li J., Yang H., Zeng H., Han W., Zhang J., Lee M., Moczygemba M., Isgandarova S., Yang Y. (2017). Mutations in 5-Methylcytosine Oxidase TET2 and RhoA Cooperatively Disrupt T Cell Homeostasis. J. Clin. Investig..

[25] Ng S.Y., Brown L., Stevenson K., deSouza T., Aster J.C., Louissaint A., Weinstock D.M. (2018). RhoA G17V Is Sufficient to Induce Autoimmunity and Promotes T-Cell Lymphomagenesis in Mice. Blood.

[26] Delfau-Larue M.-H., de Leval L., Joly B., Plonquet A., Challine D., Parrens M., Delmer A., Salles G., Morschhauser F., Delarue R. (2012). Targeting Intratumoral B Cells with Rituximab in Addition to CHOP in Angioimmunoblastic T-Cell Lymphoma. A Clinicobiological Study of the GELA. Haematologica.

[27] Lemonnier F., Safar V., Beldi-Ferchiou A., Cottereau A.-S., Bachy E., Cartron G., Fataccioli V., Pelletier L., Robe C., Letourneau A. (2021). Integrative Analysis of a Phase 2 Trial Combining Lenalidomide with CHOP in Angioimmunoblastic T-Cell Lymphoma. Blood Adv..

[28] Wulf G.G., Altmann B., Ziepert M., D’amore F., Held G., Greil R., Tournilhac O., Relander T., Viardot A., Wilhelm M. (2021). Alemtuzumab plus CHOP versus CHOP in Elderly Patients with Peripheral T-Cell Lymphoma: The DSHNHL2006-1B/ACT-2 Trial. Leukemia.

[29] Bachy E., Camus V., Thieblemont C., Sibon D., Casasnovas R.-O., Ysebaert L., Damaj G., Guidez S., Pica G.M., Kim W.S. (2022). Romidepsin Plus CHOP Versus CHOP in Patients With Previously Untreated Peripheral T-Cell Lymphoma: Results of the Ro-CHOP Phase III Study (Conducted by LYSA). J. Clin. Oncol..

[30] Pro B., Horwitz S.M., Prince H.M., Foss F.M., Sokol L., Greenwood M., Caballero D., Morschhauser F., Wilhelm M., Iyer S.P. (2017). Romidepsin Induces Durable Responses in Patients with Relapsed or Refractory Angioimmunoblastic T-Cell Lymphoma. Hematol. Oncol..

[31] Mohammed Saleh M.F., Kotb A., Abdallah G.E.M., Muhsen I.N., El Fakih R., Aljurf M. (2021). Recent Advances in Diagnosis and Therapy of Angioimmunoblastic T Cell Lymphoma. Curr. Oncol..

[32] Coiffier B., Pro B., Prince H.M., Foss F., Sokol L., Greenwood M., Caballero D., Borchmann P., Morschhauser F., Wilhelm M. (2012). Results from a Pivotal, Open-Label, Phase II Study of Romidepsin in Relapsed or Refractory Peripheral T-Cell Lymphoma after Prior Systemic Therapy. J. Clin. Oncol..

[33] Zhang W., Su L., Liu L., Gao Y., Wang Q., Su H., Song Y., Zhang H., Shen J., Jing H. (2021). The Combination of Chidamide with the CHOEP Regimen in Previously Untreated Patients with Peripheral T-Cell Lymphoma: A Prospective, Multicenter, Single Arm, Phase 1b/2 Study. Cancer Biol. Med..

[34] O’Connor O.A., Horwitz S., Masszi T., Van Hoof A., Brown P., Doorduijn J., Hess G., Jurczak W., Knoblauch P., Chawla S. (2015). Belinostat in Patients With Relapsed or Refractory Peripheral T-Cell Lymphoma: Results of the Pivotal Phase II BELIEF (CLN-19) Study. J. Clin. Oncol..

[35] Ghione P., Faruque P., Mehta-Shah N., Seshan V., Ozkaya N., Bhaskar S., Yeung J., Spinner M.A., Lunning M., Inghirami G. (2020). T Follicular Helper Phenotype Predicts Response to Histone Deacetylase Inhibitors in Relapsed/Refractory Peripheral T-Cell Lymphoma. Blood Adv..

[36] Lemonnier F., Dupuis J., Sujobert P., Tournillhac O., Cheminant M., Sarkozy C., Pelletier L., Marçais A., Robe C., Fataccioli V. (2018). Treatment with 5-Azacytidine Induces a Sustained Response in Patients with Angioimmunoblastic T-Cell Lymphoma. Blood J. Am. Soc. Hematol..

[37] Ruan J., Moskowitz A.J., Mehta-Shah N., Sokol L., Chen Z., Rahim R., Song W., Van Besien K., Horwitz S.M., Rutherford S.C. (2020). Multi-Center Phase II Study of Oral Azacitidine (CC-486) Plus CHOP As Initial Treatment for Peripheral T-Cell Lymphoma (PTCL). Blood.

[38] Falchi L., Ma H., Klein S., Lue J.K., Montanari F., Marchi E., Deng C., Kim H.A., Rada A., Jacob A.T. (2021). Combined Oral 5-Azacytidine and Romidepsin Are Highly Effective in Patients with PTCL: A Multicenter Phase 2 Study. Blood.

[39] Horwitz S.M., Koch R., Porcu P., Oki Y., Moskowitz A., Perez M., Myskowski P., Officer A., Jaffe J.D., Morrow S.N. (2018). Activity of the PI3K-δ,γ Inhibitor Duvelisib in a Phase 1 Trial and Preclinical Models of T-Cell Lymphoma. Blood.

[40] Brammer J. (2021). Duvelisib in Patients with Relapsed/Refractory Peripheral T-Cell Lymphoma from the Phase 2 Primo Trial: Results of an Interim Analysis.

[41] Mondragón L., Mhaidly R., De Donatis G.M., Tosolini M., Dao P., Martin A.R., Pons C., Chiche J., Jacquin M., Imbert V. (2019). GAPDH Overexpression in the T Cell Lineage Promotes Angioimmunoblastic T Cell Lymphoma through an NF-ΚB-Dependent Mechanism. Cancer Cell.

[42] Colell A., Ricci J.-E., Tait S., Milasta S., Maurer U., Bouchier-Hayes L., Fitzgerald P., Guio-Carrion A., Waterhouse N.J., Li C.W. (2007). GAPDH and Autophagy Preserve Survival after Apoptotic Cytochrome c Release in the Absence of Caspase Activation. Cell.

[43] Colell A., Green D.R., Ricci J.-E. (2009). Novel Roles for GAPDH in Cell Death and Carcinogenesis. Cell Death Differ..

[44] Cildir G., Low K.C., Tergaonkar V. (2016). Noncanonical NF-ΚB Signaling in Health and Disease. Trends Mol. Med..

[45] Myles A., Cancro M.P. (2016). The NIK of Time for B Cells. Eur. J. Immunol..

[46] Miyawaki S., Nakamura Y., Suzuka H., Koba M., Yasumizu R., Ikehara S., Shibata Y. (1994). A New Mutation, Aly, That Induces a Generalized Lack of Lymph Nodes Accompanied by Immunodeficiency in Mice. Eur. J. Immunol..

[47] Li Y., Wang H., Zhou X., Xie X., Chen X., Jie Z., Zou Q., Hu H., Zhu L., Cheng X. (2016). Cell Intrinsic Role of NF-ΚB-Inducing Kinase in Regulating T Cell-Mediated Immune and Autoimmune Responses. Sci. Rep..

[48] Xiao G., Harhaj E.W., Sun S.C. (2001). NF-KappaB-Inducing Kinase Regulates the Processing of NF-KappaB2 P100. Mol. Cell.

[49] Cheng J., Feng X., Li Z., Zhou F., Yang J.-M., Zhao Y. (2022). Pharmacological Inhibition of NF-ΚB-Inducing Kinase (NIK) with Small Molecules for the Treatment of Human Diseases. RSC Med. Chem..

[50] Gray C.M., Remouchamps C., McCorkell K.A., Solt L.A., Dejardin E., Orange J.S., May M.J. (2014). Noncanonical NF-ΚB Signaling Is Limited by Classical NF-ΚB Activity. Sci. Signal..

[51] Bram R.J. (2012). TBK1 Suppression of IgA in the NIK of Time. Nat. Immunol..

[52] Jin J., Xiao Y., Chang J.-H., Yu J., Hu H., Starr R., Brittain G.C., Chang M., Cheng X., Sun S.-C. (2012). The Kinase TBK1 Controls IgA Class Switching by Negatively Regulating Noncanonical NF-ΚB Signaling. Nat. Immunol..

[53] Iqbal J., Weisenburger D.D., Greiner T.C., Vose J.M., McKeithan T., Kucuk C., Geng H., Deffenbacher K., Smith L., Dybkaer K. (2010). Molecular Signatures to Improve Diagnosis in Peripheral T-Cell Lymphoma and Prognostication in Angioimmunoblastic T-Cell Lymphoma. Blood.

[54] Liang P.-I., Chang S.-T., Lin M.-Y., Hsieh Y.-C., Chu P.-Y., Chen C.-J., Lin K.-J., Jung Y.-C., Hwang W.-S., Huang W.-T. (2014). Angioimmunoblastic T-Cell Lymphoma in Taiwan Shows a Frequent Gain of ITK Gene. Int. J. Clin. Exp. Pathol..

[55] Feldman A.L., Sun D.X., Law M.E., Novak A.J., Attygalle A.D., Thorland E.C., Fink S.R., Vrana J.A., Caron B.L., Morice W.G. (2008). Overexpression of Syk Tyrosine Kinase in Peripheral T-Cell Lymphomas. Leukemia.

[56] Smith-Garvin J.E., Koretzky G.A., Jordan M.S. (2009). T Cell Activation. Annu. Rev. Immunol..

[57] Fujisawa M., Sakata-Yanagimoto M., Nishizawa S., Komori D., Gershon P., Kiryu M., Tanzima S., Fukumoto K., Enami T., Muratani M. (2018). Activation of RHOA-VAV1 Signaling in Angioimmunoblastic T-Cell Lymphoma. Leukemia.

[58] Rohr J., Guo S., Huo J., Bouska A., Lachel C., Li Y., Simone P.D., Zhang W., Gong Q., Wang C. (2016). Recurrent Activating Mutations of CD28 in Peripheral T-Cell Lymphomas. Leukemia.

[59] Ohmoto A., Fuji S. (2019). Cyclosporine for Angioimmunoblastic T-Cell Lymphoma: A Literature Review. Expert Rev. Hematol..

[60] Boddicker R.L., Razidlo G.L., Dasari S., Zeng Y., Hu G., Knudson R.A., Greipp P.T., Davila J.I., Johnson S.H., Porcher J.C. (2016). Integrated Mate-Pair and RNA Sequencing Identifies Novel, Targetable Gene Fusions in Peripheral T-Cell Lymphoma. Blood.

[61] Liu Y., Wang X., Deng L., Ping L., Shi Y., Zheng W., Lin N., Wang X., Tu M., Xie Y. (2019). ITK Inhibition Induced in Vitro and in Vivo Anti-Tumor Activity through Downregulating TCR Signaling Pathway in Malignant T Cell Lymphoma. Cancer Cell Int..

[62] Pechloff K., Holch J., Ferch U., Schweneker M., Brunner K., Kremer M., Sparwasser T., Quintanilla-Martinez L., Zimber-Strobl U., Streubel B. (2010). The Fusion Kinase ITK-SYK Mimics a T Cell Receptor Signal and Drives Oncogenesis in Conditional Mouse Models of Peripheral T Cell Lymphoma. J. Exp. Med..

[63] Dierks C., Adrian F., Fisch P., Ma H., Maurer H., Herchenbach D., Forster C.U., Sprissler C., Liu G., Rottmann S. (2010). The ITK-SYK Fusion Oncogene Induces a T-Cell Lymphoproliferative Disease in Mice Mimicking Human Disease. Cancer Res..

[64] Lechner K.S., Neurath M.F., Weigmann B. (2020). Role of the IL-2 Inducible Tyrosine Kinase ITK and Its Inhibitors in Disease Pathogenesis. J. Mol. Med..

[65] Zhong Y., Dong S., Strattan E., Ren L., Butchar J.P., Thornton K., Mishra A., Porcu P., Bradshaw J.M., Bisconte A. (2015). Targeting Interleukin-2-Inducible T-Cell Kinase (ITK) and Resting Lymphocyte Kinase (RLK) Using a Novel Covalent Inhibitor PRN694. J. Biol. Chem..

[66] Dubovsky J.A., Beckwith K.A., Natarajan G., Woyach J.A., Jaglowski S., Zhong Y., Hessler J.D., Liu T.-M., Chang B.Y., Larkin K.M. (2013). Ibrutinib Is an Irreversible Molecular Inhibitor of ITK Driving a Th1-Selective Pressure in T Lymphocytes. Blood.

[67] Kumar A., Vardhana S., Moskowitz A.J., Porcu P., Dogan A., Dubovsky J.A., Matasar M.J., Zhang Z., Younes A., Horwitz S.M. (2018). Pilot Trial of Ibrutinib in Patients with Relapsed or Refractory T-Cell Lymphoma. Blood Adv..

[68] Yoo H.Y., Kim P., Kim W.S., Lee S.H., Kim S., Kang S.Y., Jang H.Y., Lee J.-E., Kim J., Kim S.J. (2016). Frequent CTLA4-CD28 Gene Fusion in Diverse Types of T-Cell Lymphoma. Haematologica.

[69] Lee G.J., Jun Y., Jeon Y.K., Lee D., Lee S., Kim J. (2022). Mice Transgenic for Human CTLA4-CD28 Fusion Gene Show Proliferation and Transformation of ATLL-like and AITL-like T Cells. Oncoimmunology.

[70] Nguyen T.B., Sakata-Yanagimoto M., Fujisawa M., Nuhat S.T., Miyoshi H., Nannya Y., Hashimoto K., Fukumoto K., Bernard O.A., Kiyoki Y. (2020). Dasatinib Is an Effective Treatment for Angioimmunoblastic T-Cell Lymphoma. Cancer Res..

[71] Kridin K., Ahmed A.R. (2020). Post-Rituximab Immunoglobulin M (IgM) Hypogammaglobulinemia. Autoimmun. Rev..

[72] Stone E.L., Pepper M., Katayama C.D., Kerdiles Y.M., Lai C.-Y., Emslie E., Lin Y.C., Yang E., Goldrath A.W., Li M.O. (2015). ICOS Coreceptor Signaling Inactivates the Transcription Factor FOXO1 to Promote Tfh Cell Differentiation. Immunity.

[73] Weber J.P., Fuhrmann F., Feist R.K., Lahmann A., Al Baz M.S., Gentz L.-J., Vu Van D., Mages H.W., Haftmann C., Riedel R. (2015). ICOS Maintains the T Follicular Helper Cell Phenotype by Down-Regulating Krüppel-like Factor 2. J. Exp. Med..

[74] Warnatz K., Bossaller L., Salzer U., Skrabl-Baumgartner A., Schwinger W., van der Burg M., van Dongen J.J.M., Orlowska-Volk M., Knoth R., Durandy A. (2006). Human ICOS Deficiency Abrogates the Germinal Center Reaction and Provides a Monogenic Model for Common Variable Immunodeficiency. Blood.

[75] Shi J., Hou S., Fang Q., Liu X., Liu X., Qi H. (2018). PD-1 Controls Follicular T Helper Cell Positioning and Function. Immunity.

[76] Good-Jacobson K.L., Szumilas C.G., Chen L., Sharpe A.H., Tomayko M.M., Shlomchik M.J. (2010). PD-1 Regulates Germinal Center B Cell Survival and the Formation and Affinity of Long-Lived Plasma Cells. Nat. Immunol..

[77] Han L., Liu F., Li R., Li Z., Chen X., Zhou Z., Zhang X., Hu T., Zhang Y., Young K. (2014). Role of Programmed Death Ligands in Effective T-Cell Interactions in Extranodal Natural Killer/T-Cell Lymphoma. Oncol. Lett..

[78] Fiore D., Cappelli L.V., Broccoli A., Zinzani P.L., Chan W.C., Inghirami G. (2020). Peripheral T Cell Lymphomas: From the Bench to the Clinic. Nat. Rev. Cancer.

[79] Wartewig T., Kurgyis Z., Keppler S., Pechloff K., Hameister E., Öllinger R., Maresch R., Buch T., Steiger K., Winter C. (2017). PD-1 Is a Haploinsufficient Suppressor of T Cell Lymphomagenesis. Nature.

[80] Rauch D.A., Conlon K.C., Janakiram M., Brammer J.E., Harding J.C., Ye B.H., Zang X., Ren X., Olson S., Cheng X. (2019). Rapid Progression of Adult T-Cell Leukemia/Lymphoma as Tumor-Infiltrating Tregs after PD-1 Blockade. Blood.

[81] Barta S.K., Zain J., MacFarlane A.W., Smith S.M., Ruan J., Fung H.C., Tan C.R., Yang Y., Alpaugh R.K., Dulaimi E. (2019). Phase II Study of the PD-1 Inhibitor Pembrolizumab for the Treatment of Relapsed or Refractory Mature T-Cell Lymphoma. Clin. Lymphoma Myeloma Leuk.

[82] Lesokhin A.M., Ansell S.M., Armand P., Scott E.C., Halwani A., Gutierrez M., Millenson M.M., Cohen A.D., Schuster S.J., Lebovic D. (2016). Nivolumab in Patients With Relapsed or Refractory Hematologic Malignancy: Preliminary Results of a Phase Ib Study. J. Clin. Oncol..

[83] Neuwelt A., Al-Juhaishi T., Davila E., Haverkos B. (2020). Enhancing Antitumor Immunity through Checkpoint Blockade as a Therapeutic Strategy in T-Cell Lymphomas. Blood Adv..

[84] Zhang L., Mai W., Jiang W., Geng Q. (2020). Sintilimab: A Promising Anti-Tumor PD-1 Antibody. Front. Oncol..

[85] Guo Y.M., Liu X.F., Jiao L.J., Yin S.Y., Wang Z., Li X.X., Ma Z.P., Yang J.M., He M.X. (2019). Angioimmunoblastic T-cell lymphoma: Histopathological grading and prognosis. Zhonghua Bing Li Xue Za Zhi.

[86] Feng X., Guo W., Wang Y., Li J., Zhao Y., Qu L., Yan X., Li J., Guo Q., Young K.H. (2022). The Short-Term Efficacy and Safety of Brentuximab Vedotin Plus Cyclophosphamide, Epirubicin and Prednisone in Untreated PTCL: A Real-World, Retrospective Study. Adv. Ther..

[87] Sabattini E., Pizzi M., Tabanelli V., Baldin P., Sacchetti C.S., Agostinelli C., Zinzani P.L., Pileri S.A. (2013). CD30 Expression in Peripheral T-Cell Lymphomas. Haematologica.

[88] Salles G., Barrett M., Foà R., Maurer J., O’Brien S., Valente N., Wenger M., Maloney D.G. (2017). Rituximab in B-Cell Hematologic Malignancies: A Review of 20 Years of Clinical Experience. Adv. Ther..

[89] Brodfuehrer J., Rankin A., Edmonds J., Keegan S., Andreyeva T., Lawrence-Henderson R., Ozer J., Gao H., Bloom L., Boisvert A. (2014). Quantitative Analysis of Target Coverage and Germinal Center Response by a CXCL13 Neutralizing Antibody in a T-Dependent Mouse Immunization Model. Pharm. Res..

[90] Bhamidipati K., Silberstein J.L., Chaichian Y., Baker M.C., Lanz T.V., Zia A., Rasheed Y.S., Cochran J.R., Robinson W.H. (2020). CD52 Is Elevated on B Cells of SLE Patients and Regulates B Cell Function. Front. Immunol..

[91] Jiang L., Yuan C.M., Hubacheck J., Janik J.E., Wilson W., Morris J.C., Jasper G.A., Stetler-Stevenson M. (2009). Variable CD52 Expression in Mature T Cell and NK Cell Malignancies: Implications for Alemtuzumab Therapy. Br. J. Haematol..

[92] Buckstein R., Fraser G., Cheung M., Kukreti V., Kuruvilla J., Imrie K., Piliotis E., Pond G., Windsor J., Ghorab Z. (2016). Alemtuzumab and CHOP Chemotherapy for the Treatment of Aggressive Histology Peripheral T Cell Lymphomas: A Multi-Center Phase I Study. Clin. Lymphoma Myeloma Leuk.

[93] Krug A., Martinez-Turtos A., Verhoeyen E. (2021). Importance of T, NK, CAR T and CAR NK Cell Metabolic Fitness for Effective Anti-Cancer Therapy: A Continuous Learning Process Allowing the Optimization of T, NK and CAR-Based Anti-Cancer Therapies. Cancers.

[94] Park J.H., Rivière I., Gonen M., Wang X., Sénéchal B., Curran K.J., Sauter C., Wang Y., Santomasso B., Mead E. (2018). Long-Term Follow-up of CD19 CAR Therapy in Acute Lymphoblastic Leukemia. N. Engl. J. Med..

[95] Breman E., Demoulin B., Agaugué S., Mauën S., Michaux A., Springuel L., Houssa J., Huberty F., Jacques-Hespel C., Marchand C. (2018). Overcoming Target Driven Fratricide for T Cell Therapy. Front. Immunol..

[96] Scarfò I., Ormhøj M., Frigault M.J., Castano A.P., Lorrey S., Bouffard A.A., van Scoyk A., Rodig S.J., Shay A.J., Aster J.C. (2018). Anti-CD37 Chimeric Antigen Receptor T Cells Are Active against B- and T-Cell Lymphomas. Blood.

[97] Pinz K.G., Yakaboski E., Jares A., Liu H., Firor A.E., Chen K.H., Wada M., Salman H., Tse W., Hagag N. (2017). Targeting T-Cell Malignancies Using Anti-CD4 CAR NK-92 Cells. Oncotarget.

[98] Maciocia P.M., Wawrzyniecka P.A., Philip B., Ricciardelli I., Akarca A.U., Onuoha S.C., Legut M., Cole D.K., Sewell A.K., Gritti G. (2017). Targeting the T Cell Receptor β-Chain Constant Region for Immunotherapy of T Cell Malignancies. Nat. Med..

[99] Pearce E.J., Pearce E.L. (2018). Immunometabolism in 2017: Driving Immunity: All Roads Lead to Metabolism. Nat. Rev. Immunol..

[100] Chang C.-H., Curtis J.D., Maggi L.B., Faubert B., Villarino A.V., O’Sullivan D., Huang S.C.-C., van der Windt G.J.W., Blagih J., Qiu J. (2013). Posttranscriptional Control of T Cell Effector Function by Aerobic Glycolysis. Cell.

[101] Xu Y., Chaudhury A., Zhang M., Savoldo B., Metelitsa L.S., Rodgers J., Yustein J.T., Neilson J.R., Dotti G. (2016). Glycolysis Determines Dichotomous Regulation of T Cell Subsets in Hypoxia. J. Clin. Investig..

[102] Stauss D., Brunner C., Berberich-Siebelt F., Höpken U.E., Lipp M., Müller G. (2016). The Transcriptional Coactivator Bob1 Promotes the Development of Follicular T Helper Cells via Bcl6. EMBO J..

[103] Basso K., Dalla-Favera R. (2012). Roles of BCL6 in Normal and Transformed Germinal Center B Cells. Immunol. Rev..

[104] Manso R., Martínez-Magunacelaya N., Chamizo C., Rojo F., Piris M.Á., Rodriguez-Pinilla S.M. (2018). Mutual Regulation between BCL6 and a Specific Set of MiRNAs Controls TFH Phenotype in Peripheral T-Cell Lymphoma. Br. J. Haematol..

[105] Bunting K.L., Melnick A.M. (2013). New Effector Functions and Regulatory Mechanisms of BCL6 in Normal and Malignant Lymphocytes. Curr. Opin. Immunol..

[106] Nishizawa S., Sakata-Yanagimoto M., Hattori K., Muto H., Nguyen T., Izutsu K., Yoshida K., Ogawa S., Nakamura N., Chiba S. (2017). BCL6 Locus Is Hypermethylated in Angioimmunoblastic T-Cell Lymphoma. Int. J. Hematol..

[107] Leeman-Neill R.J., Bhagat G. (2018). BCL6 as a Therapeutic Target for Lymphoma. Expert Opin. Ther. Targets.

[108] Cardenas M.G., Oswald E., Yu W., Xue F., MacKerell A.D., Melnick A.M. (2017). The Expanding Role of the BCL6 Oncoprotein as a Cancer Therapeutic Target. Clin. Cancer Res..

[109] Kerres N., Steurer S., Schlager S., Bader G., Berger H., Caligiuri M., Dank C., Engen J.R., Ettmayer P., Fischerauer B. (2017). Chemically Induced Degradation of the Oncogenic Transcription Factor BCL6. Cell Rep..

[110] Parry R.V., Chemnitz J.M., Frauwirth K.A., Lanfranco A.R., Braunstein I., Kobayashi S.V., Linsley P.S., Thompson C.B., Riley J.L. (2005). CTLA-4 and PD-1 Receptors Inhibit T-Cell Activation by Distinct Mechanisms. Mol. Cell Biol..

[111] Klein Geltink R.I., O’Sullivan D., Corrado M., Bremser A., Buck M.D., Buescher J.M., Firat E., Zhu X., Niedermann G., Caputa G. (2017). Mitochondrial Priming by CD28. Cell.

[112] Waickman A.T., Powell J.D. (2012). MTOR, Metabolism, and the Regulation of T-Cell Differentiation and Function. Immunol. Rev..

[113] Patsoukis N., Bardhan K., Chatterjee P., Sari D., Liu B., Bell L.N., Karoly E.D., Freeman G.J., Petkova V., Seth P. (2015). PD-1 Alters T-Cell Metabolic Reprogramming by Inhibiting Glycolysis and Promoting Lipolysis and Fatty Acid Oxidation. Nat. Commun..

[114] Scharping N.E., Menk A.V., Moreci R.S., Whetstone R.D., Dadey R.E., Watkins S.C., Ferris R.L., Delgoffe G.M. (2016). The Tumor Microenvironment Represses T Cell Mitochondrial Biogenesis to Drive Intratumoral T Cell Metabolic Insufficiency and Dysfunction. Immunity.

[115] MacIver N.J., Michalek R.D., Rathmell J.C. (2013). Metabolic Regulation of T Lymphocytes. Annu. Rev. Immunol..

[116] Mahadevan D., Spier C., Della Croce K., Miller S., George B., Riley C., Warner S., Grogan T.M., Miller T.P. (2005). Transcript Profiling in Peripheral T-Cell Lymphoma, Not Otherwise Specified, and Diffuse Large B-Cell Lymphoma Identifies Distinct Tumor Profile Signatures. Mol. Cancer Ther..

[117] Xiong J., Bian J., Wang L., Zhou J.-Y., Wang Y., Zhao Y., Wu L.-L., Hu J.-J., Li B., Chen S.-J. (2015). Dysregulated Choline Metabolism in T-Cell Lymphoma: Role of Choline Kinase-α and Therapeutic Targeting. Blood Cancer J..

[118] Bachow S.H., O’Connor O.A. (2015). Emerging Therapies in Relapsed and Refractory Peripheral T-Cell Lymphoma. Clin. Adv. Hematol. Oncol..

[119] Poirier F., Joubert-Caron R., Labas V., Caron M. (2003). Proteomic Analysis of a Lymphoma-Derived Cell Line (DG75) Following Treatment with a Demethylating Drug: Modification of Membrane-Associated Proteins. Proteomics.

